# A novel dataset of guava fruit for grading and classification

**DOI:** 10.1016/j.dib.2023.109462

**Published:** 2023-07-28

**Authors:** Abdul Khalique Maitlo, Abdul Aziz, Hassnian Raza, Neelam Abbas

**Affiliations:** Department of Computer Science SZABIST Larkana, Larkana Pakistan

**Keywords:** Guava fruit, Grading, Machine learning, Deep Learning

## Abstract

Machine learning algorithms play a vital role in object detection and recognition. Currently, Machine learning techniques have achieved significant performance in various areas. However, there is still a need for research in the agriculture sector. The fruit harvesting process is carried out by unskilled labour without using modern scientific technologies; resultantly, the accuracy of harvesting is compromised. Moreover, immature fruits were harvested, which caused revenue losses and pretended sustainable growth. Therefore, the classification and grading of fruits are increasingly highlighted amongst the research communities. This article presents a novel dataset for local varieties such as Local Sindhi, Thadhrami and Riyali of guava fruit harvested in the Larkana region of Pakistan. The dataset is a primary instrument for developing an autonomous system using machine learning and deep learning methods. Hence, it has come up with an indigenous and state-of-the-art dataset. The dataset was developed using varieties as mentioned above. The dataset has been classified into three folders; each folder was further divided into three subfolders related to maturity level (i) Green, (ii) Mature Green, and (iii) Ripe. Images have been acquired in a controlled environment. The proposed dataset contains 2,309 total images in jpg format. This dataset will contribute to developing machine learning-based systems for the agricultural sector.

**Specifications Table**

**Table:** 

Subject	Computer Science, agriculture science
Specific subject area	Image processing, machine learning
Data format	Raw images having jpg format
Type of data	Images of guava fruit
Data collection	The dataset was collected using a Canon EOS 5D Mark III camera. All the images were captured at the same light illumination and distance. Furthermore, images were resized to 850 × 1300.
Data source location	Location: Village Choohar Pur, Tehsil (Taluka) Naudero, District Larkana. City: Larkana Province: Sindh Country: Pakistan
Data accessibility	Repository name: Mendeley Data Data identification number:10.17632/w3fg8jjmzr.1 Direct URL of Dataset: https://data.mendeley.com/datasets/w3fg8jjmzr

## Value of the Data

• This novel dataset of guava fruit is used to develop an automated classification system in the industry to overcome the classification challenge.
• Fruit processing industries and researchers can utilize datasets to develop a service-orientated platform for farmers to identify maturity levels and strengthen quality production systems.
• The guava fruit dataset increases production quality during the industry's grading and classification process. Various models can be trained and tested to maximize the system's accuracy.
• The collection of datasets consists of various steps like fruit collection with the help of experts, arranging a controlled environment to take images from the top view, assigning labels according to class and scaling all images with equal height and width like other existing datasets [1], [2], [3]. This dataset strengthens agricultural research.
• Recognition of varieties of guava fruit and maturity levels boost the country's economic growth.

## 1 Objective

To develop a system for the industry which utilize for grading and classification. The proposed dataset is used to construct a machine-learning model that improves quality production.

## 2 Data Description

The photographed image dataset was classified through two essential classification elements: the variety of fruit and the maturity stage. The acquired dataset images were stored in the guava dataset folder. It was further classified into three sub-folders named (Local Sindhi, Thadhrami and Riyali) variety. Moreover, the variety folder contains three sub-folders based on maturity level (Green, Mature Green & Ripe). The image acquisition process is defined in Fig. 2. Each fruit class contains images like Riyali 655, Local Sindhi 711 and Thadhrami 943.

**Fig 1:**
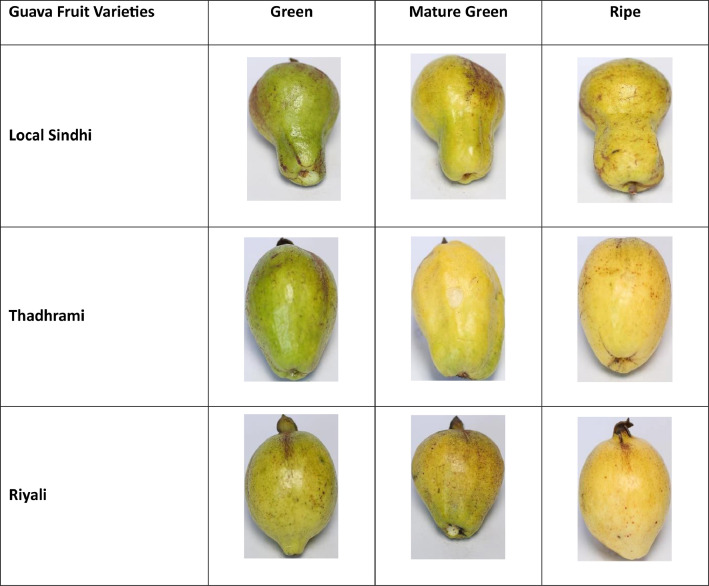
Sample of dataset images.

Additionally, the distribution of dataset images is depicted in Table 1. The guava fruit is classified and graded through colour, shape, size, and texture. The sample images are shown in Fig. 1, according to varieties and maturity levels.

**Table 1:** Guava fruit dataset details.

Guava Varieties	Green	Mature Green	Ripe	Total
Local Sindhi	98	360	253	711
Riyali	67	101	487	655
Thadhrami	67	368	508	943
			Grand Total	2,309

**Fig 2:**
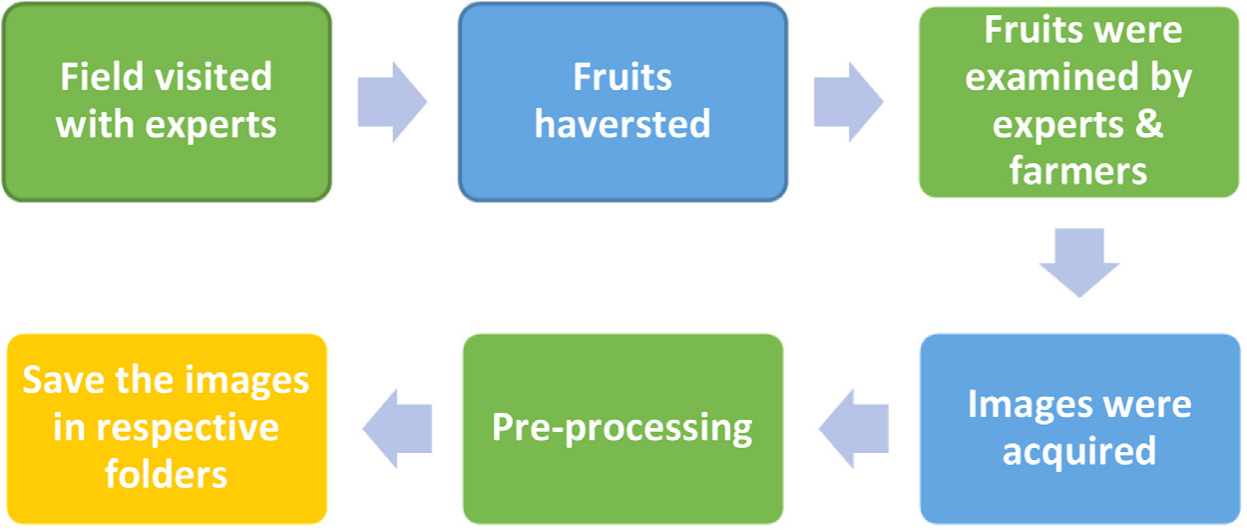
Process of dataset preparation.

## 3 Experimental Design, Materials and Methods

The guava fruit was collected from Shahani field, village Choohar Pur Naudero Road, Larkana, in December 2022. The maturity stage and variety identification were accomplished with the assistance of local farmers and experts. The 250 random sample fruits were harvested of every variety at each maturity stage. Experts and local farmers, with their expertise and years of experience, examined the harvested fruit of varieties. They recommend only 200 fruits for image acquisition.

The dataset images were acquired through a Canon EOS 5D Mark III camera of 22.3 megapixels, and the exposure time was 1/160 s. Other settings remained the default. The acquired images were stored in their folders according to variety and growth stage. The stored dataset was used for pre-processing.

(i) The photographed images were shortlisted based on clarity, blurriness and affirmed for further process. (ii) Labels were assigned to each image. (iii) Edges of fruit were detected using a canny edge detector. (iv) The morphological operations were performed, and the region of interest (ROI) was extracted. (v) After extraction of ROI, a new image was created in jpg format and stored in the respective folder.

The selected fruits were brought into a controlled environment, where all the necessary arrangements were made. Images were captured using a high-definition Canon EOS 5D Mark III camera at the same angle, colour, background, and light. The distance between the camera and the guava fruit was approximately 87 cm.

The original size of the acquired images was 3840 × 5760. Moreover, images were resized to 850 × 1300 pixels using Python programming.

## Ethics Statement

In the process of dataset preparation, both did not imply the utilization of human subjects or involve any experiments on animals.

## CRediT authorship contribution statement

**Abdul Khalique Maitlo:** Methodology, Data curation, Writing – review & editing, Investigation, Validation. **Abdul Aziz:** Formal analysis. **Hassnian Raza:** Project administration. **Neelam Abbas:** Writing – original draft, Data curation.

## Data Availability

Guava Fruit Dataset for Classification (Original data) (Mendeley Data).
